# Implications for paediatric shock management in resource-limited settings: a perspective from the FEAST trial

**DOI:** 10.1186/s13054-018-1966-4

**Published:** 2018-05-04

**Authors:** Kirsty Anne Houston, Elizabeth C. George, Kathryn Maitland

**Affiliations:** 1 Wellcome Trust Centre for Clinical Tropical Medicine and Department of Paediatrics, Faculty of Medicine, Imperial College, London, W2 1PG UK; 2 KEMRI-Wellcome Trust Research Programme, Centre for Geographic Medicine Research-Coast, PO Box 230, Kilifi, Kenya; 3 Medical Research Council Clinical Trials Unit (MRC CTU) at University College London (UCL), 90 High Holborn, 2nd Floor, London, WC1V 6XX UK

**Keywords:** Paediatric, Shock, World Health Organization (WHO), Tachycardia, Hypotension, Intravenous fluids

## Abstract

**Background:**

Although the African “Fluid Expansion as Supportive therapy” (FEAST) trial showed fluid resuscitation was harmful in children with severe febrile illness managed in resource-limited hospitals, the most recent evidence reviewed World Health Organization (WHO) guidelines continue to recommend fluid boluses in children with shock according to WHO criteria “WHO shock”, arguing that the numbers included in the FEAST trial were too small to provide reasonable certainty.

**Methods:**

We re-analysed the FEAST trial results for all international definitions for paediatric shock including hypotensive (or decompensated shock) and the WHO criteria. In addition, we examined the clinical relevance of the WHO criteria to published and unpublished observational studies reporting shock in resource-limited settings.

**Results:**

We established that hypotension was rare in children with severe febrile illness complicating only 29/3170 trial participants (0.9%). We confirmed that fluid boluses were harmful irrespective of the definitions of shock including the very small number with WHO shock (n = 65). In this subgroup 48% of bolus recipients died at 48 h compared to 20% of the non-bolus control group, an increased absolute risk of 28%, but translating to an increased relative risk of 240% (*p* = 0.07 (two-sided Fisher’s exact test)). Examining studies describing the prevalence of the stringent WHO shock criteria in children presenting to hospital we found this was rare (~ 0.1%) and in these children mortality was very high (41.5–100%).

**Conclusions:**

The updated WHO guidelines continue to recommend boluses for a very limited number of children presenting at hospital with the strict definition of WHO shock. Nevertheless, the 3% increased mortality from boluses seen across FEAST trial participants would also include this subgroup of children receiving boluses. Recommendations aiming to differentiate WHO shock from other definitions will invariably lead to “slippage” at the bedside, with the potential of exposing a wider group of children to the harm of fluid-bolus therapy.

**Electronic supplementary material:**

The online version of this article (10.1186/s13054-018-1966-4) contains supplementary material, which is available to authorized users.

## Background

In adult sepsis the primary definition of shock almost invariably involves hypotension as a prerequisite [1]. In children however, the majority of paediatric guidelines do not include hypotension as an essential criterion [2–5]. The only exception to this are the Advanced Paediatric Life Support (APLS) guidelines where shock is rationalised into compensated and decompensated shock, with only the latter characterised by the presence of hypotension [2] (summarised in Table 1). The term “compensated shock” is therefore more widely accepted in paediatric practice and is synonymous with severely impaired perfusion. Hypotension or decompensated shock is purported to be a rare and late feature in children, and associated with a high mortality rate. Yet, evidence to support this contention from systematic, unselected observational data from children is lacking. Worldwide, these definitions have been integrated into guidelines to direct acute management of critically unwell children.

**Table 1:** Paediatric shock definitions

Guideline	Clinical definition	Limitations
Advanced Paediatric Life Support (APLS)	Compensated: normal blood pressure (BP), but capillary refill time (CRT) >2 s, mottled, cool peripheries, peripheral cyanosis. Decompensated: as above but with hypotension, decreased mental status	Definition of hypotension is separate to shock definition Peripheral cyanosis is rare
American Academy of Critical Care Medicine – Paediatric Advanced Life Support (ACCM-PALS)	Clinical signs of inadequate perfusion including any of: decreased or altered mental status; CRT >2 s (cold shock) or flash CRT (warm shock), diminished (cold shock) or bounding (warm shock) peripheral pulses, mottled cool extremities (cold shock), or decreased urine output (<1 ml/kg/h)	No specific definition of altered/decreased mental status
World Health Organization (WHO)	Triad of cold hands and/or feet (temperature gradient), CRT >3 s and weak and fast pulse	Tachycardia not defined alongside shock definition
Fluid Expansion As A Supportive Therapy (FEAST) study	History of fever and temperature ≥37.5 °C or <36.0 °C and impaired consciousness (prostration or coma) and/or respiratory distress Stratum A (impaired perfusion) Plus ≥1 of: CRT >2 s; lower limb temperature gradient; weak pulse; tachycardia (defined) Stratum B (decompensated shock) Systolic BP <50 mmHg if < 12 months old; <60 mmHg if 1–5 years old; <70 mmHg if > 5 years old	

The absolute criteria defining paediatric shock vary from guideline to guideline, and include some poorly specified parameters, in general including similar clinical signs of “impaired perfusion”, such as capillary refill time (CRT), temperature gradient and assessment of pulse volume. Whilst each of these signs are individually associated with a poor outcome in univariate and multivariate analyses, many have yet to be formally validated by the relevant physiological studies against evidence of macro-vascular and micro-vascular compromise [6–8]. This is particularly true of the paediatric shock definition used by the World Health Organization (WHO), which has been adapted from international guidelines for low-resource hospitals, and is thus relevant to large parts of the world [5]. For children, the criteria for WHO shock includes the presence of all three signs of impaired perfusion, that is a weak and fast pulse, cool peripheries plus a CRT >3 s. Ultimately, irrespective of the definition, identifying children with shock is the first step in implementing immediate care, for which fluid resuscitation has been the cornerstone of management.

The “Fluid Expansion as Supportive therapy” (FEAST) trial (detailed in “Methods”) was designed to inform guidelines on fluid resuscitation in severe febrile illness in resource-limited settings [9]. The FEAST trial, published in 2011, showed that fluid resuscitation was harmful and led to excess mortality in all subgroups [9, 10] and across all definitions of shock [11]. The most recent review and the recommendations of the technical expert group that drew up the WHO Emergency Triage and Treatment guidelines (for resource-limited settings) in 2016 continue to recommend fluid boluses in children with shock as defined by the WHO (“WHO shock”) maintaining that, whilst the direction of harm was entirely consistent with the overall analysis, the numbers in this subgroup of the FEAST trial were too small to provide reasonable certainty [12]. We therefore have revisited the FEAST trial results and applied these to all international definitions of paediatric shock, with critical emphasis on WHO shock. Furthermore, since the FEAST trial identified very few children with WHO shock, we reviewed the applicability of the WHO shock definition to published data from admission or emergency departments reporting shock and outcome in resource-limited hospitals.

## Methods

The phase III FEAST trial was a multi-centre, randomized, controlled trial in 3141 children at six hospitals across East Africa, designed to assess the effects of fluid resuscitation in critically unwell children in resource-limited settings [9]. The FEAST trial included children with severe febrile illness and shock. Severe febrile illness was defined as history of fever or recorded fever with either impaired consciousness (defined as inability to sit unsupported or inability to localise a painful stimulus) or respiratory distress (defined as increased work of breathing (intercostal indrawing or deep acidotic breathing) or both. Children with gastroenteritis, severe malnutrition, burns or surgical conditions were excluded.

The FEAST trial considered two strata for shock: FEAST A (compensated shock) included children with one or more of the following: CRT >2 s; lower limb temperature gradient; weak pulse; heart rate (HR) >180 (age <12 months), >160 (age 12 months–5 years), >140 (age >5 years). FEAST B included children with decompensated shock with severe hypotension (defined as systolic blood pressure (SBP) <50 mmHg if age was < 2 months; <60 mmHg if age was 1−5 years; <70 mmHg if age was > 5 years).

The eligibility criteria for FEAST A were informed by a critical review of international shock criteria and applied to the Kilifi Clinical Surveillance as the reference dataset in order to inform a high-risk population for the FEAST trial. Since 1989 the Kenya Medical Research Institute (KEMRI) Programme has prospectively recorded ward admission and discharge data using a standardised proforma to systematically document clinical admission data on all infants and children entering the hospital wards. Since 2002 this has been linked to demographic surveillance in the district [13]. For this analysis we included over 14,000 unselected paediatric admissions (aged over 28 days) to Kilifi District Hospital (KDH), excluding children with severe malnutrition, at the point of triage.

First, we presented the results of the FEAST trial using this trial definition of shock and compared this with other international definitions of shock (Table 1). Outcomes for those with severe hypotension (FEAST B) were presented, but since the FEAST trial criteria for hypotension were more stringent than other guidelines (Additional file 1: Table S1) [2, 4], we investigated outcome with a less strict definition (based on other international guideline criteria) and whether this could identify a high-risk group benefiting from fluid bolus therapy [10]. Moderately severe hypotension was therefore defined as SBP of 50–75, 60–75 and 70–85 mmHg, respectively, in children aged < 12 months, 1–5 years and > 5 years.

Second, we modeled the risk of death by 48 h after admission according to subgroups of children with the different combination of features of impaired perfusion and estimated the relative risk with bolus versus no bolus using Poisson regression (with robust standard errors). Finally, to examine the relevance of WHO shock to other populations we undertook a literature and narrative search to identify papers reporting on WHO shock in children presenting to hospital and their outcome.

We did not publish a protocol for the literature search prior to conducting this review. A search of online literature was performed, including PubMed/Medline, Global Health Library, Cochrane Database of Systematic Reviews, Cochrane Central Register of Controlled Trials, ClinicalTrials.gov and the WHO International Clinical Trials Registry. There were predetermined criteria for the eligibility of studies and data outcomes. The selection criteria included children aged 0–12 years admitted to hospital with all or any features of shock (defined above) and the primary outcome was mortality. English search terms were used. We included observational and randomised controlled trials.

## Results

**Fig. 1:**
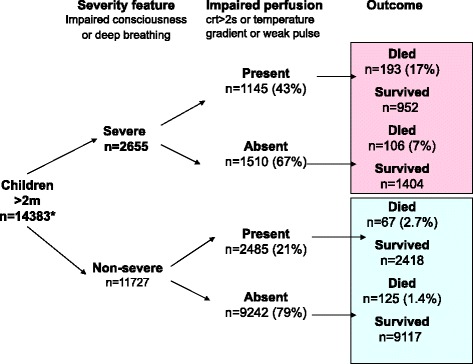
Validating the FEAST trial shock criteria in a general paediatric admission cohort. *Excludes children with severe malnutrition. CRT, capillary refill time; m, months

To identify clinical criteria determining high-risk groups with severe febrile illness and impaired perfusion for the future FEAST trial we initially stratified the KDH admission cohort according to clinical signs of severity (the presence of impaired consciousness and/or increased work of breathing). The second stratification used any one of the three signs of severely impaired perfusion (shock); CRT ≥3 s, weak pulse volume or a temperature gradient (Fig. 1). First, this demonstrates that children with any signs of clinical severity are at much higher risk of fatal outcome. Second, children at the highest risk were those with severity features accompanied by signs of impaired perfusion (17% mortality). In the absence of severity features the presence of any sign of impaired perfusion was associated with a much lower mortality (2.7%). These data therefore informed the inclusion criteria for the FEAST trial and definitions of shock (FEAST A).

**Fig. 2:**
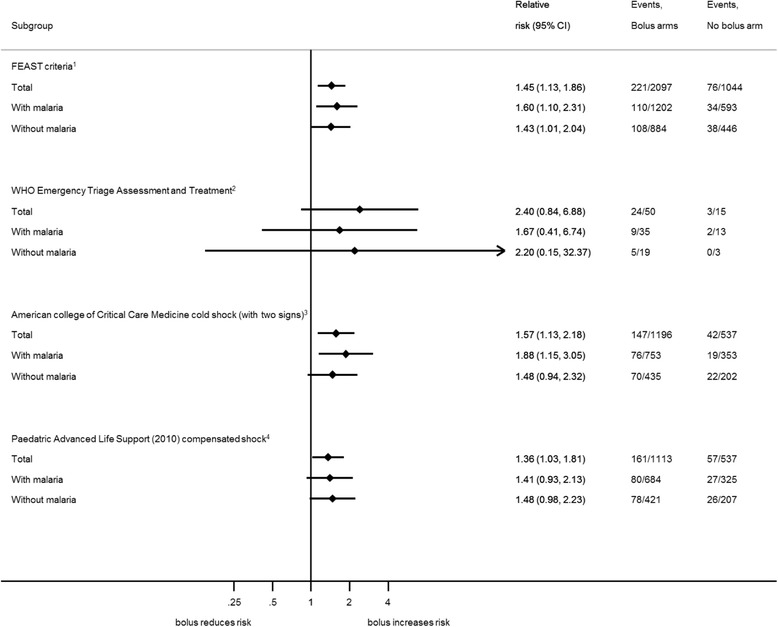
Forest plot comparing outcome with fluid bolus therapy in all shock definition (using data derived from the FEAST dataset). Note: there are 16 children with missing malaria results who are not included in the calculations in children with/without malaria. ^1^FEAST trial criteria: history of fever or axillary temperature >37.4 °C or <36 °C with impaired consciousness (prostration or coma) or respiratory distress, plus ≥ 1 of the following: capillary refill time >2 s, lower limb temperature gradient, weak pulse, tachycardia (heart rate >180 beats per min (bpm) (age <12 months), >160 bpm (age 12 months−5 years), >140 bpm (age >5 years)). ^2^World Health Organization (WHO) Emergency Triage Assessment Treatment criteria: the presence of cold hands or feet with capillary refill time >3 s and a weak pulse. ^3^American College of Critical Care Medicine (ACCM) cold shock (with two signs): axillary temperature >37.4 °C or <36 °C plus ≥ 2 of: prostration/coma or Blantyre coma score <5, capillary refill time >2 s, weak pulse, increased temperature gradient. ^4^Paediatric Advanced Life Support (PALS) (2010) compensated shock: two of the following: tachycardia (see FEAST criteria for definition), increased temperature gradient, capillary refill time >2 s, weak pulse

Applying the results of the FEAST trial to all published definitions of paediatric shock (Fig. 2) shows a remarkable consistency of harm from bolus resuscitation, resulting in a worse outcome compared with no-bolus control across all definitions. Shock, as defined by the WHO, applied to only 65 (2%) of the 3141 children in the FEAST A stratum with 24/50 (48%) who received boluses dying within 48 h, compared to only 3/15 (20%) of control children - a relative risk increase of 240% (*p* = 0.07 (two-sided Fisher’s exact test)). The risk of death by 48 h and risk ratio for bolus versus no bolus according to the number of features of impaired perfusion indicated no evidence that excess 48-h mortality in the FEAST trial differed with the number of signs of impaired perfusion (*p* = 0.34) (Additional file 2: Table S2). There was also no evidence that the excess risk of death associated with bolus fluid differed by the presence or absence of each individual sign (Additional file 3: Table S3). The impact of bolus fluid within these subgroups was consistent with the overall results of the trial.

### Severe hypotension

Of the 7838 children screened, 3170 children were enrolled into the FEAST trial (FEAST A and B), but only 29 (0.9%) fulfilled the definition of severe hypotension (FEAST B definition), confirming that hypotension is rare in children with clinically defined shock. Hypotensive shock was associated with a very poor outcome. In the FEAST B stratum (who all received fluid bolus therapy) the overall mortality was 62%. Of interest, only 8/29 (27.5%) hypotensive children fulfilled the WHO shock definition and all these participants died.

### Moderately severe hypotension

Further sub-analyses were performed in children included in the FEAST A stratum, using less stringent criteria for moderately hypotensive children. Only 72/3141 (2.3%) children in the FEAST A stratum fulfilled this definition and this was associated with higher overall mortality of 26%. Consistent with the overall analysis, fluid bolus therapy in children with moderate hypotension was not beneficial compared to no-bolus controls (RR = 1.48, 95% CI 0.61–3.66, *p* = 0.41) but this was not statistically significant owing to small numbers.

### Clinical relevance of WHO shock in practice?

We identified six observational studies reporting features of paediatric shock [14–17]. Through personal communication, we obtained a sub-analysis of two published data sets from cohorts totaling over 20,000 children admitted to two coastal hospitals in Kenya (Table 2). One was conducted at Coast Provincial General Hospital incorporating 26,104 children aged < 6 years. Only 27 children had WHO shock recorded at admission (0.1%) and this was associated with a high case fatality rate of 85%. The second was a re-analysis of a larger, prospective, observational, admission dataset from KDH, from which we originally derived the FEAST shock score. This included 22,911 children aged < 6 years. We found that only 33 children fulfilled WHO shock criteria at triage (0.14%) and this was associated a case fatality rate of 58%. Across all of the identified databases and published data, most reported that WHO shock was rare (~ 0.1% of admissions). WHO shock was consistently associated with a very high mortality rate (41.5–100%) in all of these study cohorts, where reported.

**Table 2:** Frequency of children presenting with signs of impaired circulation or shock to hospital in low-resource settings

Reference	Study design	Study site	Sample	Inclusion criteria	WHO shock and mortality	Number with ≥ 2 signs of impaired circulation
Tamburlini, 1999	Prospective cohort	Brazil	3837	Children 7 days to 5 years old presenting to emergency room	4 (0.13%) 100% mortality	ETAT emergency signs (severe respiratory distress, shock, coma/convulsions or severe dehydration) in 98 children
Robertson, 2001	Prospective cohort	QEQH Blantyre, Malawi	2281	Emergency room triage Children aged < 5 years	Not reported	Emergencies (n = 92); only 7–11 had delayed CRT (staff differed in assessments)
Ahmad, 2010	Prospective cohort study	QECH Blantyre, Malawi	583	“Critically ill” children presenting to emergency room	Did not report WHO shock triad	247 (42%)
Maitland, 2011	Phase III RCT	6 hospitals Kenya, Uganda and Tanzania	3141	FEAST trial inclusion criteria	65 (2%) 41.5% mortality	3076 (98%) by inclusion criteria
Mbevi, 2016,	Retrospective analysis	14 hospitals Kenya	42,937	Admissions in children aged > 30 days to < 5 years (excluded patients with burns or malnutrition)	41 (0.1%) Mortality not reported	3219 (7.5%)^a^
CPGH, 2017, unpublished	Prospective cohort	CPGH, Mombasa, Kenya	26,104	Admissions <=6 years over 6 years	27 (0.1%) 85% mortality	3403 (13.04%) – mortality 31%
KDH, 2017, unpublished	Prospective cohort	KDH, Kilifi, Kenya	22,911	Admissions <=6 years over 6 years	33 (0.14%) 58% mortality	9788 (42.72%) – mortality 7.24%

ETAT Emergency Triage, Assessment And Treatment, *WHO* World Health Organization, *CRT* capillary refill time, *KDH* Kilifi District Hospital, *CPGH* Coast Provincial General Hospital, *QECH* Queen Elizabeth Central Hospital

^a^Shock-associated mortality was more broadly defined: a clinician’s indication that the child had shock as a problem accompanying diarrhoea and dehydration (an indication of the severity of fluid loss); a diagnosis of shock associated with an underlying cause (e.g. septic shock); or use of rapid bolus fluid therapy in a child irrespective of diagnosis

## Discussion

To address the debate about whether children meeting the FEAST trial shock or impaired perfusion criteria [18–20] were relevant to other international shock definitions, we re-analysed the FEAST trial data. We have demonstrated that irrespective of the definition of shock, including those with moderate hypotension, fluid bolus therapy led to an increased risk of mortality in children with severe febrile illness admitted to low-resource hospitals. In addition, we were able to assess the utility of hypotension in children with assumed septic shock as the FEAST trial data probably represents the most comprehensive assessment of this parameter since the trial permitted the sickest children to be enrolled through a deferred consent process [21], and as all children had measurement of baseline blood pressure. We found that only 29 children (0.9%) fulfilled the definition of severe hypotension (FEAST B definition) confirming that hypotension is rare in children with clinically defined shock and was associated with a very poor outcome (62% mortality among all those receiving fluid bolus therapy) [9]. Nevertheless, this may have been context specific in hospitals that have no formal pre-hospital paramedic referral teams.

Three systematic reviews of fluid bolus therapy in resource-limited hospitals have been conducted since the publication of the FEAST trial [12, 22, 23]. Two were conducted by the WHO guideline group [12] and Opiyo et al. [23], and these were undertaken to inform international and Kenyan guidelines, respectively. These latter two studies included a separate sub-analysis of the very small subgroup of children meeting the WHO shock criteria. Owing to the small sample size in this sub-group, and inferred “indirectness” of this evidence, this resulted in strong or conditional recommendations for the use of fluid resuscitation, contrasting to the recommendations from the systematic review by Ford et al. [22]. We further consider these recommendations with references to the Grading of Recommendations Assessment, Development and Evaluation (GRADE) guidance [24] which has important implications for guideline recommendations in general that go beyond the interpretation of the FEAST trial data.

Our first consideration is a strong practical one. The WHO guidelines continue to advise that boluses are useful in children with all three features of shock, despite the evidence underpinning these recommendations having been rated as “conditional” based on a GRADE systematic review noting a “low quality of evidence” to support this. Our review indicates that the number and thus relevance to clinical practice of children actually fulfilling these criteria are extremely limited. In practice, the difference between the WHO and other definitions of shock comes down to capillary refill time (CRT) and cold peripheries (defined as cold hands and feet rather than temperature gradient), which have inherent difficulties in interpretation with notable inter-observer variation in assessment [25]. The stringent analysis of WHO shock in the FEAST data found that this group made up only 2% of the 3141 children recruited in the trial. However, they were the sickest children, accounting for more than 9% of the deaths. The key question is whether this group of 65 children is big enough to provide strong evidence to support generalisation of the trial results. There are widely held beliefs that the strength of evidence of a clinical trial comes only from its size. The fact is this is not true. Strong evidence can come from smaller trials if the underlying risk in the population is high and the trial results in a large difference in outcome between the treatment strategies [26]. This was case for the group of 65 children in the FEAST trial identified by WHO criteria.

In general terms, in any clinical trial showing a difference in outcome between groups of patients given different treatments, researchers need to be sure that this is caused by the intervention being tested, and that it is not the result of chance. Only then can the trial result be deemed statistically significant - and the result be generalised with a reasonable degree of confidence. There are two inter-related factors to determine this. The first is the size of the difference between the groups or arms in the trial. The second is the numbers of patients involved. If the difference between arms is large, then a small number of patients are required to confirm statistical significance. If however, the difference in outcomes is small, then larger numbers of patients are needed.

So how does this relate to FEAST? Overall, there was only a 3% absolute difference between the bolus and no-bolus groups, which was statistically significant after the fifth interim analysis when trial recruitment was halted prematurely with 3141 participants enrolled [27]. The recommendation of the data monitoring committee, at this point, was that even if the trial recruited the projected sample size (n = 3200), it would never be able show a benefit of fluid boluses. The main result indicated a 3% overall increase in harm associated with fluid bolus therapy; however, relevant to WHO recent recommendations, in the group of 65 children with WHO-defined shock (a group with much higher risk of death), the difference is stark. Almost half, 48%, of the children given boluses died compared to 20% of those not given boluses, an increased absolute risk of 28% [11]. As the numbers are small one cannot be sure the 28% difference is accurate; however, as this difference is so large, occurring across multiple centres, and is in the same direction as the main result, one can say with a great deal of confidence that children in the WHO group will suffer some degree of harm from fluid boluses. Indeed, one can say with 95% confidence that the 3% increased mortality from boluses seen across the FEAST trial can be generalised, and would occur if all children with WHO definition shock were given boluses. This is strong evidence of harm, even though the number of children in the FEAST trail who satisfied the WHO definition of shock is small. As indicated by Yusef et al. “the overall trial result is usually a better guide to the direction of effect in subgroups than the apparent effect observed within a subgroup” [28].

The GRADE guideline committee suggests that when examining the quality of evidence, inferring “indirectness of evidence” to downgrade results should occur only when there are substantial differences exist between the populations. This should be supported by a plausible biological rationale that the subgroup differs substantially from the overall trial population. Alternatively, there is good evidence that the effect in that sub-population is significantly different from the overall population [29]. Neither of these applies to the small group with WHO shock in the FEAST trial. Whilst the WHO guidelines have made strong recommendations for children with some signs of impaired perfusion, advising that they should not receive fluid boluses (rated as a strong level of evidence), for those with WHO shock the updated guidelines recommended 10–20 ml of fluid bolus over 30–60 min (with one repeat of 10 ml/kg body weight) (Table 3). This recommendation was rated by the technical group as conditional and based on a low quality of evidence. In contrast, GRADE guidance warns against strong or conditional recommendations when confidence in effect estimates is low or very low, suggesting that such recommendations are seldom justified [29].

**Table 3:** World Health Organization Emergency Triage and Training fluid resuscitation guidelines

	Definition	Clinical management
Shock	All of (i) cold extremities and (ii) capillary refill time more than 3 s and (iii) weak and (iv) fast pulse^a^	10–20 ml/kg body weight over 30–60 min, then a further 10 ml/kg over 30 min
Impaired circulation	One or two of the three features of shock, but not the complete triad	No boluses

^a^No specific values given for tachycardia ranges

WHO shock considered a Triad of signs yet the definition indicates that four features are required

## Conclusions

The updated 2016 WHO guidelines continue to recommend fluid boluses in a very rare group of children presenting to hospital with severe febrile illness children, which is complicated by the very narrow definition of shock, despite harm being shown in this subgroup in the FEAST trial. This group represents a very small fraction of children admitted to hospital (less than 0.2%) who have a very poor outcome. By maintaining strong recommendations for use of boluses in this clinically irrelevant group (< 0.2% of admissions with extremely high mortality), we are concerned that children are placed at risk of harm from bolus therapy, with few likely to receive benefit. Moreover, any set of guidelines that separates the WHO shock definition from other shock definitions will invariably lead to confusion and “slippage” at the bedside with the potential to cause harm to a wider group of children.

## Additional files

Additional file 1: **Table S1.** Defining paediatric hypotension (mmHg). (DOCX 18 kb)

Additional file 2: **Table S2.** FEAST trial: mortality by 48 h and risk ratio for bolus versus no bolus according to the number of features of impaired perfusion (IP). (DOCX 14 kb)

Additional file 3: **Table S3.** FEAST data: risk ratio of death for bolus versus no bolus according to the presence or absence of individual shock signs. (DOCX 13 kb)

## Abbreviations

ACCM-PALS: American College of Critical Care Medicine Paediatric Advanced Life Support
ALSG: Advanced Life Support Group
APLS: Advanced Paediatric Life Support
BP: Blood Pressure
CPGH: Coast Provincial General Hospital
CRT: Capillary refill time
ED: Emergency department
ETAT: Emergency triage, assessment and treatment
FEAST: Fluid Expansion As Supportive Therapy
GRADE: Grading of Recommendations Assessment, Development and Evaluation
GSC: Guidelines Steering Committee
ICU: Intensive care unit
KDH: Kilifi District Hospital
MAP: Mean arterial pressure
MRC: Medical Research Council
PEWS: Paediatric Early Warning Score
QECH: Queen Elizabeth Central Hospital
RCH: Royal Children’s Hospital, Melbourne
RCT: Randomised controlled trial
WHO: World Health Organization
