# Supplementary material for: Implications for paediatric shock management in resource-limited settings: a perspective from the FEAST trial
Source: Crit Care. 2018 May 4;22:119. doi: 10.1186/s13054-018-1966-4 (PMC5936024; doi:10.1186/s13054-018-1966-4)
Supplement: Supplementary file 1 — Table S1. Defining paediatric hypotension (mmHg). (DOCX 18 kb) [file 13054_2018_1966_MOESM1_ESM.docx]

**Additional file 1: Table S1** Defining paediatric hypotension (mmHg)

|  | APLS[1] | ACCM-PALS^a [^2] | WHO[3] | FEAST  Stratum B ^b^ |
| --- | --- | --- | --- | --- |
| Based upon | Expert opinion (working group) | Extrapolated from adult studies | Expert opinion | Review of evidence |
| Systolic Blood pressure cut offs | | | | |
| <1yr | <70 | <60 | <60 | <50 |
| 1-5yrs | <80 | <65 | <70 (1-3yr) | <60 |
| >5yrs | <90 | <65 | <75 (3-6yr) | <70 |

**Footnote**

^a^ACCM-PALS – Mean arterial pressure references

^b^ Severe Hypotension

**References for this table**

1. **Advanced Paediatric Life Support: A Practical Approach to Emergencies**, 6th edn: Wiley-Blackwell; 2016.

2. Brierley J, Carcillo JA, Choong K, Cornell T, Decaen A, Deymann A, Doctor A, Davis A, Duff J, Dugas MA *et al*: **Clinical practice parameters for hemodynamic support of pediatric and neonatal septic shock: 2007 update from the American College of Critical Care Medicine**. *Critical care medicine* 2009, **37**(2):666-688.

3. **Pocket book of hospital care for children: Second edition Guidelines for the management of common childhood illnesses**. Geneva: World Health Organization; 2013.
