# Supplementary material for: Implications for paediatric shock management in resource-limited settings: a perspective from the FEAST trial
Source: Crit Care. 2018 May 4;22:119. doi: 10.1186/s13054-018-1966-4 (PMC5936024; doi:10.1186/s13054-018-1966-4)
Supplement: Supplementary file 2 — Table S2. FEAST trial: mortality by 48 h and risk ratio for bolus versus no bolus according to the number of features of impaired perfusion (IP). (DOCX 14 kb) [file 13054_2018_1966_MOESM2_ESM.docx]

**Additional file 2: Table S2.** FEAST trial: Mortality by 48 hours and risk ratio for bolus versus no bolus according to the number of features impaired perfusion (IP).

| **No. of**  **IP signs** | **Bolus** | | **No bolus** | | **Risk ratio (95% CI)** |
| --- | --- | --- | --- | --- | --- |
|  | **N** | **Died (%)** | **N** | **Died (%)** |  |
| **1** | 983 | 60 (6.1%) | 507 | 19 (3.7%) | 1.63 (0.98 – 2.70) |
| **2** | 678 | 68 (10.0%) | 351 | 24 (6.8%) | 1.46 (0.94 – 2.29) |
| **3 or 4** | 433 | 93 (21.5%) | 186 | 33 (17.7%) | 1.21 (0.84 – 1.73) |
