# Supplementary material for: Implications for paediatric shock management in resource-limited settings: a perspective from the FEAST trial
Source: Crit Care. 2018 May 4;22:119. doi: 10.1186/s13054-018-1966-4 (PMC5936024; doi:10.1186/s13054-018-1966-4)
Supplement: Supplementary file 3 — Table S3. FEAST data: risk ratio of death for bolus versus no bolus according to the presence or absence of individual shock signs. (DOCX 13 kb) [file 13054_2018_1966_MOESM3_ESM.docx]

**Additional file 3: Table S3.** FEAST data: Risk ratio of death for bolus versus no bolus according to the presence or absence of individual shock signs

| **Impaired perfusion sign** | **Risk ratio (Bolus vs no Bolus)** | | **P^a^** | **Adjusted risk ratio^b^**  **(95% CI)** |
| --- | --- | --- | --- | --- |
|  | **(Sign present)** | **(Sign absent)** |  |  |
| Severe tachycardia | 1.28 (0.91–1.79) | 1.66 (1.15–2.41) | 0.30 | 1.44 (1.12 – 1.85) |
| Weak pulse | 1.20 (0.86–1.65) | 1.65 (1.15–2.38) | 0.19 | 1.41 (1.10 – 1.80) |
| CRT > 2s | 1.34 (0.95–1.91) | 1.47 (1.04–2.09) | 0.72 | 1.41 (1.10 – 1.81) |
| Temperature Gradient | 1.42 (1.07–1.89) | 1.48 (0.88 – 2.50) | 0.90 | 1.44 (1.12 – 1.84) |

1. P-value for heterogeneity of risk ratio (in the presence vs absence of sign)
2. Overall risk ratio for bolus vs no bolus (assuming no heterogeneity)
